# Systematic Review and Meta-Analysis of the Frequency of Thromboembolic Events, Bleeding, and Mortality in Patients with Atrial Fibrillation and End-Stage Renal Disease Undergoing Percutaneous Left Atrial Appendage Closure

**DOI:** 10.3390/jcm15072641

**Published:** 2026-03-31

**Authors:** Juan Manuel Martínez-Arango, Laura María Rojas-Echavarría, Carolina García-Mejía, Juan Daniel Castrillón-Spitia, Luis Felipe Higuita-Gutiérrez

**Affiliations:** 1 Internal Medicine, School of Medicine, Medellín–Envigado Campus, Universidad Cooperativa de Colombia, Medellín 050026, Colombia; 2 Medicine, School of Medicine, Medellín Campus, Universidad CES, Medellín 050021, Colombia; 3 Research Unit, School of Medicine, Medellín–Envigado Campus, Universidad Cooperativa de Colombia, Medellín 050026, Colombia; luis.higuita@campusucc.edu.co

**Keywords:** atrial fibrillation, renal insufficiency, thrombosis, left atrial appendage, anticoagulation

## Abstract

**Background**: Atrial fibrillation (AF) and end-stage renal disease (ESRD) are closely related conditions that increase the risk of disability, stroke, and mortality. Anticoagulation management in patients with ESRD and AF is challenging due to the high risk of bleeding. Percutaneous left atrial appendage closure (LAAC) has emerged as an alternative to reduce thromboembolic events; however, evidence in this specific population remains limited. Therefore, we aimed to evaluate the frequency of thromboembolic events, bleeding complications and mortality in patients with AF and ESRD undergoing LAAC through a systematic review and meta-analysis. **Methods**: A systematic review and meta-analysis were conducted following PRISMA 2020 guidelines and registered in PROSPERO (CRD420250640241). A structured search was performed in Medline, EMBASE, Web of Science, SCOPUS, LILACs and institutional repositories through September 2024, with no language restrictions. We included original studies reporting frequencies of thromboembolic events, bleeding and mortality in patients with AF and ESRD undergoing LAAC. A random-effects model was used and heterogeneity was assessed using the I^2^ statistic. **Results**: Fourteen studies were included in the qualitative analysis and seven in the quantitative synthesis, comprising a total of 2433 patients with AF and ESRD undergoing LAAC. In the qualitative analysis, the mean age was 74 ± 7.6 years; the most common comorbidities were hypertension (74%), diabetes mellitus (47%), and dyslipidemia (53%). Watchman™ devices predominated in North America, whereas Amulet™ devices were more frequently used in Europe and Latin America. Procedural success was 98.4%, with infrequent periprocedural complications: major bleeding in 1.6% and device embolization in 0.5%. In the quantitative analysis, the pooled frequency of thromboembolic events was 3% (95% CI: 1–7%; I^2^ = 81.1%), pooled bleeding frequency was 6% (95% CI: 4–10%; I^2^ = 76.9%), and pooled mortality was 5% (95% CI: 1–22%; I^2^ = 97.8%). After excluding studies with extreme values, adjusted mortality was 2% (95% CI: 1–5%; I^2^ = 76.6%). Despite high heterogeneity, the findings suggest that LAAC may offer protection against embolic events with an acceptable bleeding risk. **Conclusions**: LAAC in patients with AF and ESRD is associated with a low frequency of thromboembolic events and bleeding when compared with standard anticoagulation therapy and no treatment. Overall mortality is moderate and appears to be primarily attributable to underlying comorbidity rather than the procedure itself. This meta-analysis provides evidence that LAAC may be a safe and effective therapeutic strategy in patients with contraindications or high risk for chronic anticoagulation. However, prospective and comparative clinical trials are needed to confirm these findings and inform future clinical practice guidelines.

## 1. Introduction

The relationship between atrial fibrillation (AF) and chronic kidney disease (CKD) is complex and bidirectional. Both conditions confer an increased risk of disability and functional decline, and their coexistence is associated with a significantly higher mortality rate [1].

AF is the most common cardiac arrhythmia worldwide. Between 2010 and 2019, its global prevalence increased from 33.5 million to 59 million cases [2,3], reaching up to 10% in individuals older than 85 years [4]. In terms of incidence, approximately 4.72 million new cases of AF were reported globally in 2019 [5]. Projections indicate that incidence will continue to rise over the coming decades. In 2021, the age-standardized incidence rate reached 52.4 per 100,000 inhabitants, and estimates suggest that by 2035 this rate will increase to 54.89 per 100,000 inhabitants [6].

The clinical relevance of AF is reflected in its substantial impact on patients’ quality of life [7]. It is associated with up to a fivefold increase in the risk of ischemic stroke a 1.5–2-fold higher risk of death compared with the general population [8], and an increased risk of cognitive decline and dementia [2]. In older adults, AF is linked to progressive functional loss: independence in activities of daily living declines by 4.4% per year, while independence in instrumental activities of daily living decreases by 3.9% annually [9].

In parallel, CKD has been associated with adverse outcomes. For every 10 mL/min reduction in estimated glomerular filtration rate (eGFR), the risk of stroke increases by 7% [10]. This risk rises even further once AF develops [11]. Additionally, patients with CKD exhibit defects in primary hemostasis due to abnormal interactions between platelets and the endothelium. These dysfunctions arise from the presence of uremic toxins, alterations in arachidonic acid metabolism, von Willebrand factor abnormalities and decreased intracellular levels of adenosine diphosphate and serotonin. Collectively, these mechanisms markedly increase bleeding risk in this population [12].

In patients with ESRD, particularly those undergoing hemodialysis, the prevalence of AF is substantially higher, ranging from 8% to 35%, making it the most common arrhythmia in this group [13,14]. This finding has been associated with chronic inflammation and population aging [15,16]. AF in ESRD is linked to a higher risk of thromboembolic events, particularly stroke, as well as a greater incidence of bleeding complications [17,18]. Furthermore, the coexistence of ESRD and AF complicates clinical management, particularly anticoagulation, due to the heightened risk of bleeding [19,20,21,22].

Anticoagulation therapy has long been the standard approach for preventing thromboembolic events in patients with AF. However, the evidence supporting the use of oral anticoagulants, either vitamin K antagonists (VKAs) or direct oral anticoagulants (DOACs), is less robust in individuals with creatinine clearance below 30 mL/min, as many of these patients were excluded from pivotal randomized clinical trials [23]. In patients with ESRD, especially those requiring renal replacement therapy, warfarin has not demonstrated a reduction in stroke risk and is associated with an increased risk of major bleeding [21]. Although DOACs may offer safety and efficacy advantages, evidence remains limited and concerns persist regarding the high incidence of significant bleeding events [24,25].

With advances in technology and the development of minimally invasive strategies, new alternatives aimed at preventing thromboembolic events while minimizing bleeding risk have emerged. Percutaneous left atrial appendage closure (LAAC) stands out as a procedure that, through various endovascular techniques and devices, seeks to anatomically exclude the left atrial appendage, the primary site of thrombus formation, with the goal of preventing systemic embolization and its severe consequences. This intervention is considered a potentially safer therapeutic option in selected populations, particularly those at high risk of stroke and bleeding or with contraindications to long-term anticoagulation [26].

Randomized clinical trials have evaluated the safety and efficacy of this intervention [27,28]. The PROTECT AF [29] and PREVAIL [30] trials demonstrated that LAAC with the Watchman™ device was non-inferior to warfarin for preventing cerebrovascular events in patients with non-valvular AF. The PRAGUE-17 trial [26], which enrolled patients at high risk of bleeding, concluded that LAAC using either the Amplatzer Amulet™ or Watchman™ device was non-inferior to DOACs for preventing cardiovascular, neurological, and bleeding events.

Major primary studies have evaluated LAAC in patients with AF and high bleeding risk, but they excluded patients with ESRD, making it controversial to extrapolate their findings to this subgroup. Although a potential benefit of LAAC is hypothesized for preventing thromboembolic and bleeding events in patients with ESRD and AF, given their high risk of thrombosis without treatment and high bleeding risk with long-term anticoagulation [31], current evidence remains scarce, and the frequency of procedure-related outcomes in this population is largely unknown. This gap underscores the need for an exhaustive review of the available literature.

The primary objective of this study is to evaluate, through a systematic review and meta-analysis, the frequency of thromboembolic events, bleeding, and mortality in patients with AF and ESRD undergoing LAAC, compared with outcomes described in the literature for patients treated with conventional antithrombotic strategies or no anticoagulation, in order to estimate the overall clinical effects associated with the procedure.

## 2. Methods

### 2.1. Study Design

This systematic review and meta-analysis were conducted in accordance with the PRISMA 2020 guidelines, ensuring compliance with all 27 methodological items. The PRISMA 2020 checklist has been included in the supplementary materials (Table S1). The protocol was previously evaluated and approved by the International Prospective Register of Systematic Reviews (PROSPERO), under registration number CRD420250640241.

### 2.2. Search Strategy

A systematic and structured search was performed in PubMed, EMBASE, EBSCO, ScienceDirect, Web of Science, SCOPUS, LILACs, OVID, Google Scholar, and institutional repositories including the Texas Data Repository, Electrophysiology Physiome Model Repository, Radboud University Repository, and the institutional repository E-docUR of Universidad del Rosario.

The search covered all records from database inception through 30 September 2024, which represents the final date of the literature search for this systematic review. The strategy was designed and validated by a librarian specialized in systematic reviews and independently reviewed by two investigators. The general search structure was developed using MeSH terms and free-text keywords combined with Boolean operators, truncations, and field filters.

Complete electronic search strategies for all databases, including queries, filters, and execution dates, are provided in Supplementary Material Table S2 to ensure reproducibility.

All records were exported in RIS format into Rayyan™ for duplicate management and title/abstract screening.

### 2.3. Screening Process

All abstracts were reviewed independently by two investigators to assess compliance with the inclusion criteria. In cases of disagreement, a third investigator made the final decision. Selected studies were uploaded in full text into Rayyan™, where duplicate removal was automated. Subsequently, each investigator assessed studies considered non-eligible based on predefined criteria.

### 2.4. Data Extraction

All extracted variables were systematically organized into a structured Microsoft Excel database, later exported in .xlsx and .csv formats to ensure transparency and reproducibility. This dataset served as the basis for descriptive analyses and cross-checking between reviewers.

Primary efficacy outcomes included the frequency of thromboembolic events and mortality; the primary safety outcome was the frequency of bleeding from any cause. Secondary outcomes included short- and long-term procedure-related complications.

Quality assessment of included studies was performed using the STROBE checklist and the Joanna Briggs Institute (JBI) critical appraisal checklist for prevalence studies. These tools were used to construct the risk-of-bias table in RevMan 5™.

### 2.5. Study Selection

We identified manuscripts including patients with stage 5 CKD and atrial fibrillation at high embolic risk undergoing percutaneous LAAC and reporting frequencies of thromboembolic events, bleeding, and mortality. No comparator was required.

We included original articles in any language available in the consulted databases up to September 2024 that reported absolute and relative frequencies of the measured outcomes, allowing the calculation of incidence and prevalence.

Studies were required to provide numerical data on outcomes in patients undergoing LAAC with a prior diagnosis of AF and ESRD, defined as an eGFR < 15 mL/min/1.73 m^2^ (using CKD-EPI, MDRD, or Cockcroft–Gault formulas), requirement for renal replacement therapy, or identification based on clinical criteria using ICD-10 classification codes. Detailed eligibility criteria are provided in Supplementary Material Table S3.

### 2.6. Summary Measures

Findings were summarized descriptively. Categorical variables were presented as *n* (%), and continuous variables as mean ± standard deviation (SD) or median (interquartile range, IQR), depending on data distribution. For multinational studies, patient counts were evenly redistributed across countries and rounded prior to analysis.

Analyses and results were organized according to clinical characteristics, comorbidities, risk classifications, and other population-specific variables. Frequencies and proportions were evaluated together with measures of central tendency and dispersion. The pooled frequency of outcomes of interest and their 95% confidence intervals were estimated.

### 2.7. Statistical Analysis

Quantitative analyses and descriptive statistics were performed using RStudio version 4.5.2 (Posit Software, Boston, MA, USA), which was also used to generate visualizations (world maps, frequency distributions, descriptive graphs) with the ggplot2 and sf packages.

Meta-analyses for proportions were conducted to estimate cumulative effects and 95% confidence intervals using random-effects models based on inverse-variance weighting and logit transformation. Begg–Mazumdar rank correlation tests and Tau correlation coefficients were performed. Heterogeneity was assessed using the I^2^ statistic, with values > 50% considered indicative of substantial heterogeneity. Statistical significance was determined using the chi-square–based Q test, with *p* < 0.10 considered significant. Funnel plots were generated to assess publication bias. These analyses were conducted using the platform developed by Fekete & Gyorffy [32].

## 3. Results

**Figure 1:**
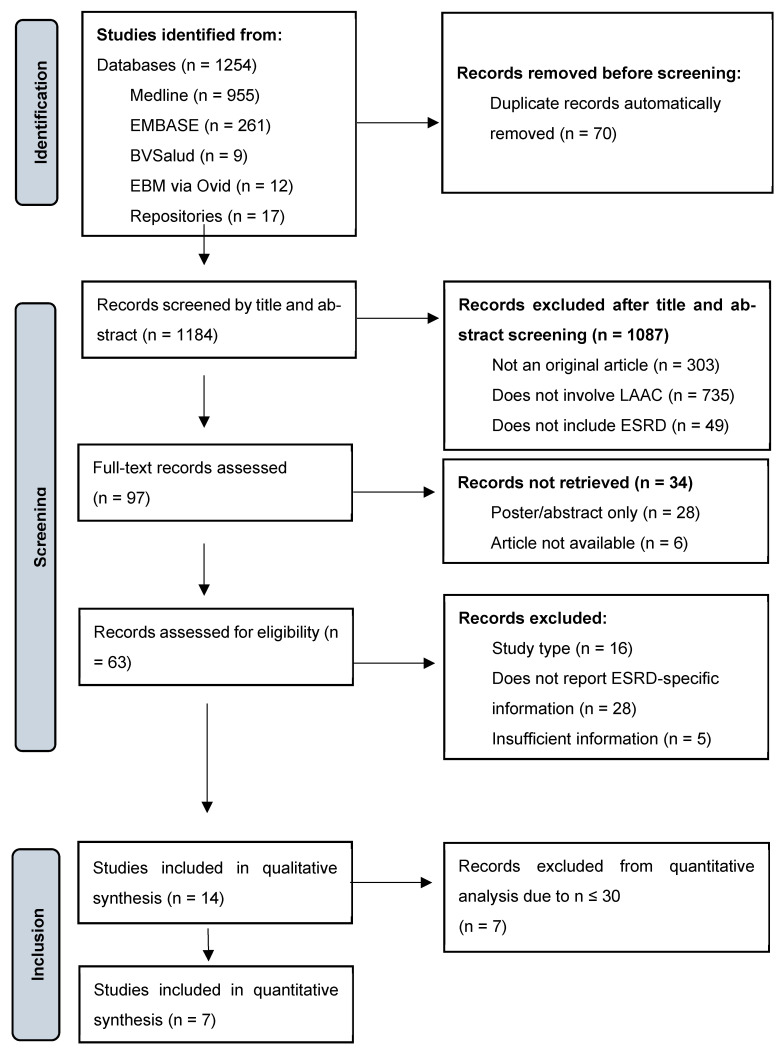
PRISMA 2020 Flow Diagram Summarizing the Study Selection Process. Flow diagram illustrating the phases of identification, screening, eligibility, and inclusion for this exploratory review on LAAC in patients with ESRD. A total of 1254 records were initially identified across five databases and repositories; after the removal of 70 duplicates and subsequent screening and eligibility assessments, 14 studies met the inclusion criteria for the qualitative synthesis. Source: Prepared by the authors based on PRISMA 2020 guidelines. Abbreviations: ESKD = end-stage kidney disease; LAAC = left atrial appendage closure.

Following the systematic search, a total of 1254 records were initially identified from databases such as Medline, EMBASE, BVSalud, EBM via Ovid, and institutional repositories. After title and abstract screening, 1087 records were excluded for the following reasons: not being original articles (*n* = 303), not addressing LAAC (*n* = 735), or not including patients with ESRD (*n* = 49). A total of 97 full-text articles were assessed, of which 34 could not be retrieved (28 posters, 6 unavailable articles), and 48 were excluded for various reasons. The final analysis included 14 studies in the qualitative synthesis; subsequently, eight studies with a sample size ≤ 30 patients were excluded, leaving a total of seven studies for the quantitative synthesis (Figure 1).

### 3.1. Qualitative Analysis

**Figure 2:**
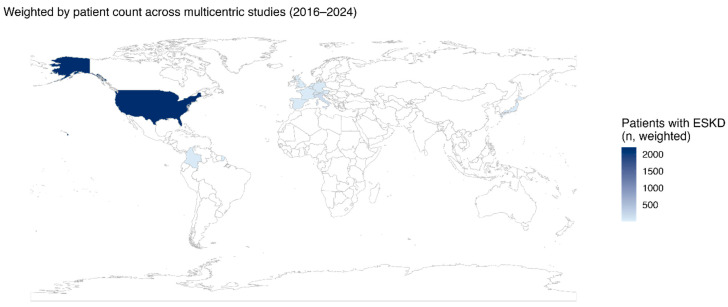
Geographic distribution of patients with ESKD undergoing LAAC. The map illustrates the patient-weighted density of studies included in the scoping review (2016–2024), showing the relative contribution (*n*) of each country to the total ESKD population analyzed. Darker shades of blue indicate a higher number of included patients per country. Abbreviations: ESKD—end-stage kidney disease; LAAC—left atrial appendage closure. Source: Authors’ elaboration in RStudio version 4.5.2 using rnaturalearth, ggplot2, and sf packages (Scoping Review 2016–2024).

A total of fourteen observational studies published between 2016 and 2024 were included, comprising 2571 patients with ESRD, defined as an estimated glomerular filtration rate (eGFR) ≤ 15 mL/min/1.73 m^2^ or dialysis dependence, and AF undergoing LAAC [33,34,35,36,37,38,39,40,41,42,43,44,45,46]. Most studies corresponded to retrospective cohorts or multicenter registries, while three were small case series. The populations analyzed originated from Europe, Asia, and the Americas, providing a broad and representative overview of LAAC in the ESRD population (Figure 2).

### 3.2. Study and Patient Characteristics

Of the 2571 patients included, 2251 (87.5%) were undergoing dialysis at the time of the procedure, predominantly hemodialysis. This distribution was homogeneous across studies, although some registries included mixed subgroups of transplanted patients or individuals with stage 5 CKD not yet on dialysis [33,40]. The population was consistently elderly, with a pooled mean age of approximately 74 ± 7.6 years. When data were reported as median and interquartile range, the central value remained around 74 years [IQR 69–79], with minimal regional variation. Larger registries showed stable mean ages between 71 and 76 years, with no evidence of selection bias across data sources [37,38,39,40,41,42,43]. The proportion of male patients was 65.7%, reflecting the typical sex distribution of the ESRD population with cardiovascular comorbidity (Table 1).

### 3.3. Baseline Comorbidities

Hypertension was the most prevalent comorbidity, affecting more than 74% of patients. Diabetes mellitus was reported in 47% and dyslipidemia in 53%. Cardiovascular diseases, including coronary artery disease, heart failure, and peripheral arterial disease, were common, with rates between 40 and 50%, underscoring the cardiovascular fragility of this population. A history of stroke or transient ischemic attack was present in 8% of patients, smoking in 11%, and obesity in 4%. Liver disease was infrequent (<5%).

### 3.4. Atrial Fibrillation Phenotype and Risk Profile

Permanent AF predominated across most cohorts, accounting for approximately 60% of cases, whereas persistent or paroxysmal AF comprised the remaining 40%. This pattern was consistent among the larger registries [33,35,38], reflecting the predominance of permanent AF in this high-risk group. Regarding risk stratification, 51.4% of patients had a CHA_2_DS_2_-VASc score ≥ 5 and 46.9% had a HAS-BLED score ≥ 4, confirming a dual profile of high thromboembolic and hemorrhagic risk characteristic of populations with advanced CKD.

### 3.5. Devices Used

**Figure 3:**
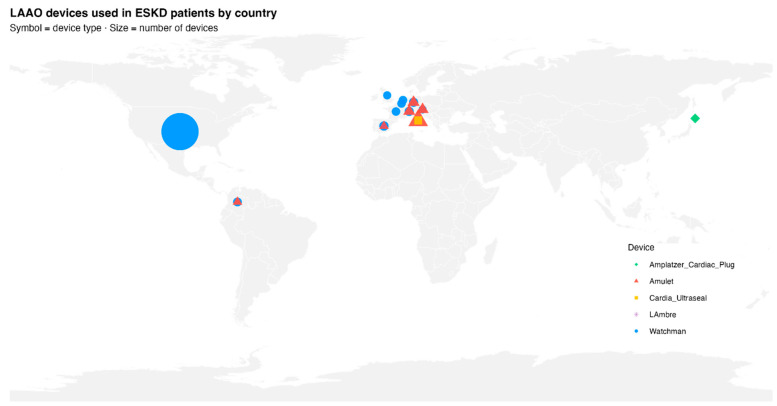
Geographical distribution of LAAC devices used in patients with AF and ESKD across included studies. Each symbol represents a device type, and the size of the symbol corresponds to the number of procedures performed per country. Blue circles denote Watchman devices, red triangles Amulet, yellow squares Cardia Ultraseal, green diamonds Amplatzer Cardiac Plug, and violet stars LAmbre. The United States contributed the largest proportion of procedures, followed by Italy, Japan, and several European countries. Abbreviations: LAAC, left atrial appendage closure; AF, atrial fibrillation; ESKD, end-stage kidney disease. Source: Authors’ elaboration using RStudio (sf, rnaturalearth, ggplot2).

A defined geographic distribution was observed (Figure 3). The Watchman™ device predominated in North American studies, accounting for approximately 2190 patients (85%), while the Amulet™ device was more frequently used in Europe and Latin America (around 316 patients, 12%) [33,34,35,36,37,38,39,40,41,42,43,44,45]. Other devices, such as LAmbre™, the Amplatzer Cardiac Plug™, and Cardia Ultraseal™, represented approximately 40 procedures (1.5%), primarily in Asian and European case series [42,46]. Despite heterogeneity in device types, procedural performance was highly consistent across studies.

### 3.6. Procedural Success

Technical success was uniformly high, with 2531 successful implants (98.4%) and minimal peridevice leak. Multicenter registries with large sample sizes reported success rates between 97% and 99%, while small single-center series achieved 100% success. This consistency supports the reproducibility and technical feasibility of LAAC, even in highly comorbid ESRD patients.

### 3.7. Periprocedural Complications

Periprocedural complications were infrequent. Device embolization occurred in 13 patients (0.5%), as reported in multicenter registries. Access-site hematomas were observed in 32 patients (1.2%), and major periprocedural bleeding in 42 patients (1.6%), most of which were reported in large cohorts [33,34,35,38]. None of the small series reported intraprocedural deaths or cardiac tamponade. Most bleeding events were minor (local hematomas or puncture-site bleeding), and no significant differences were observed between device types. Overall, LAAC in ESRD patients demonstrated high technical success and a low rate of immediate complications, even in complex, multicenter, real-world settings.

### 3.8. Follow-Up Outcomes

During follow-up, most bleeding events corresponded to gastrointestinal bleeding (2.4%), while intracranial hemorrhage accounted for less than 1% (0.6%). These rates remained consistent across regions and were independent of device type or study design. Most hemorrhagic events occurred within the first three months after implantation, coinciding with the period of temporary dual antithrombotic therapy, and declined following adjustment or discontinuation of this therapy.

Most thromboembolic events occurred within the first six months post-implantation, also corresponding to the dual therapy phase, with no evidence of an increase in late events during long-term follow-up [33,34,35,36,37,38,46]. The majority of these events were arterial, primarily stroke or myocardial infarction, representing more than 85% of all events, while venous events were rare (<10 cases). Myocardial infarction occurred in approximately 1.0%, and peripheral or venous thromboembolism remained ≤ 0.4%. No significant association was identified between the type of implanted device and the occurrence of thromboembolic events.

## 4. Quantitative Analysis

### 4.1. Mortality Frequency

**Figure 4:**
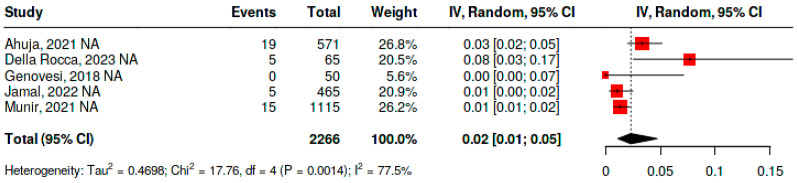
Mortality frequency in patients with atrial fibrillation and end-stage renal disease undergoing percutaneous left atrial appendage closure [33,34,36,37,39].

To assess mortality among patients undergoing percutaneous left atrial appendage closure, seven studies were included, comprising a total of 2429 patients. The analysis was performed using a random-effects model due to significant heterogeneity (*p* < 0.01) and an I^2^ of 97.8%. The pooled mortality frequency was 5% with a confidence interval (CI) ranging from 1.0% to 22%. The studies reporting the highest mortality were those by Flink et al. [35] (18%, CI 9–30%) and Genovesi et al. [38] (56%, CI 46–65%). Both studies included exclusively patients with end-stage renal disease on hemodialysis, a population with a high comorbidity burden, increased cardiovascular risk, and elevated baseline mortality; in addition, the study by Genovesi had substantially longer follow-up than the others, favoring the natural accumulation of deaths. Considering these factors, the meta-analysis was repeated excluding these two publications, and heterogeneity remained significant (*p* < 0.05), with I^2^ decreasing to 77.5%, and a resulting pooled mortality of 2% (CI 1–5%) (Figure 4).

### 4.2. Frequency of Thromboembolic Events

To evaluate the frequency of thromboembolic events in patients undergoing percutaneous left atrial appendage closure, the same 2429 patients were included. The analysis was performed using a random-effects model due to significant heterogeneity (*p* < 0.01; I^2^ = 81.2%). In this case, the pooled frequency of thromboembolic events was 3% (95% CI, 1–7%).

The study with the highest frequency of thromboembolic events was that of Fink et al. [35], reporting 9% (95% CI, 3–19%), likely because a considerable proportion of patients had ESRD, high CHA_2_DS_2_-VASc scores, and relevant comorbidities, including heart failure and prior stroke, factors that increase baseline ischemic risk even after device implantation.

**Figure 5:**
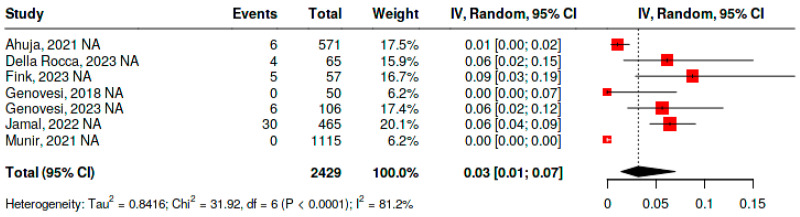
Frequency of thromboembolic events in patients with atrial fibrillation and end-stage renal disease undergoing percutaneous left atrial appendage closure [34,35,36,37,38,39].

In contrast, the studies by Genovesi et al. [39] and Munir et al. [37] reported no thromboembolic events after the intervention. Both studies had the particularity of limiting follow-up to the periprocedural period or the first 30 days, time frames during which the absolute risk of embolic events is generally low, and included smaller sample sizes and highly experienced operators, which may reduce the likelihood of early complications (Figure 5).

### 4.3. Bleeding Frequency

**Figure 6:**
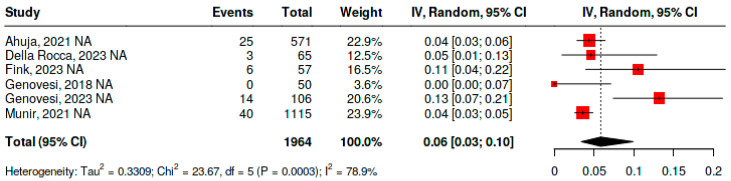
Frequency of bleeding in patients with atrial fibrillation and end-stage renal disease undergoing percutaneous left atrial appendage closure [33,34,35,37,38,39].

To evaluate the frequency of bleeding events in patients undergoing percutaneous left atrial appendage closure, six studies were included, comprising a total of 1964 patients. The analysis was performed using a random-effects model due to significant heterogeneity (*p* < 0.01; I^2^ = 78.9%). The pooled bleeding frequency was 6% (95% CI, 3–10%) (Figure 6).

The heterogeneity is mainly explained by differences in the baseline risk of the included populations and by variability in the use of dual antiplatelet therapy during the post-implantation period. Additional contributors included varying follow-up durations and differences in bleeding definitions, all of which influenced the dispersion of results.

### 4.4. Assessment of Publication Bias

Potential publication bias was assessed through visual inspection of funnel plots and the application of Egger’s test for each of the outcomes analyzed (mortality, thromboembolic events, and bleeding).

**Figure 7:**
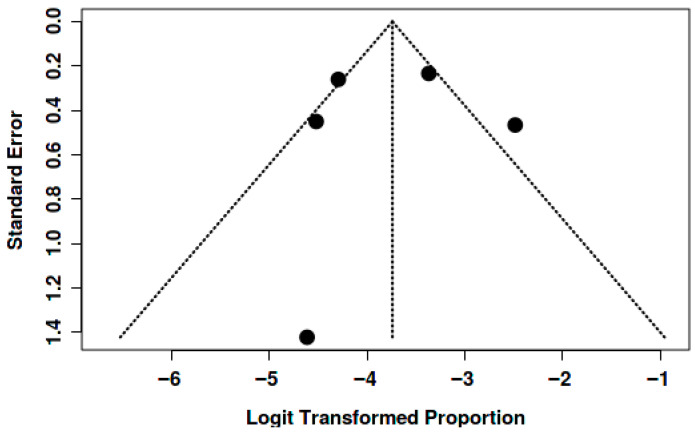
The funnel plot does not indicate a potential publication bias. The Egger’s test does not support the presence of funnel plot asymmetry (intercept: −0.24, 95% CI: −5.09–4.61, t: −0.097, *p*-value: 0.929).

For mortality, the funnel plot showed a relatively symmetrical distribution of studies around the global estimate, with no visual evidence of marked asymmetry. This was corroborated by Egger’s test, which did not indicate significant publication bias (intercept: –0.24; 95% CI: –5.09 to 4.61; *p* = 0.929). These findings suggest that the variability observed in the meta-analysis is mainly attributable to genuine clinical differences among the studied populations rather than to the absence of small or negative studies (Figure 7).

**Figure 8:**
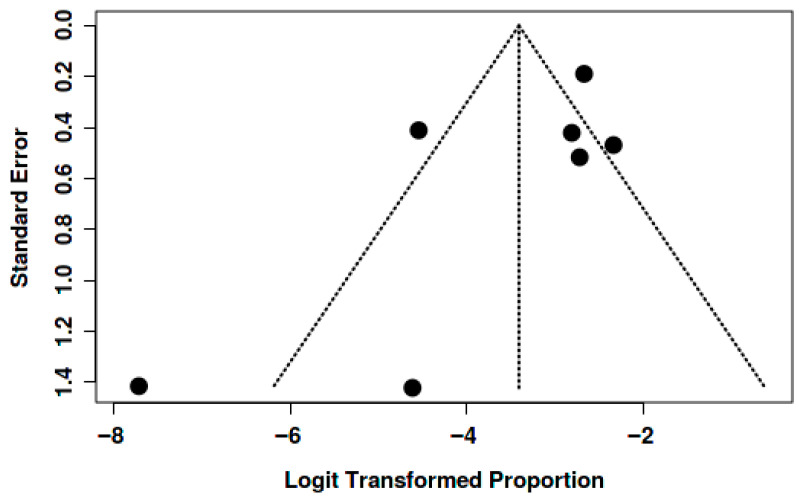
The funnel plot does not indicate a potential publication bias. The Egger’s test does not support the presence of funnel plot asymmetry (intercept: −2.33, 95% CI: −5.14–0.48, t: −1.622, *p*-value: 0.166).

In the analysis of thromboembolic events, the funnel plot also showed no systematic asymmetry. Although some dispersion was observed at the base of the plot, consistent with small sample sizes and inter-study variability, the overall distribution was compatible with the absence of relevant publication bias. Egger’s test was not statistically significant, supporting the visual interpretation (Figure 8).

**Figure 9:**
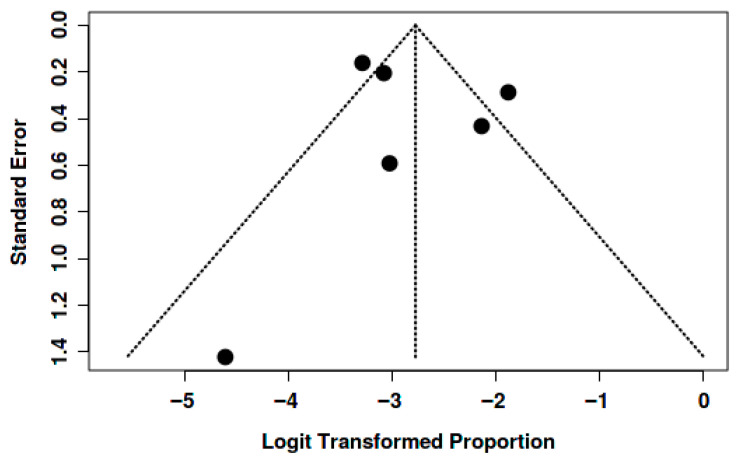
The funnel plot does not indicate a potential publication bias. The Egger’s test does not support the presence of funnel plot asymmetry (intercept: 1.01, 95% CI: −2.72–4.73, t: 0.529, *p*-value: 0.625).

Similarly, for bleeding, the funnel plot displayed a symmetrical pattern with the expected dispersion around the effect size, without consistent indications of asymmetry. Egger’s test was also non-significant (intercept: 1.01; 95% CI: –2.72 to 4.73; *p* = 0.625), suggesting no evidence of publication bias affecting the pooled estimate (Figure 9).

Overall, both graphical and statistical approaches consistently indicated no significant publication bias in the evaluated outcomes, supporting the robustness of the meta-analysis results in this regard.

### 4.5. Risk of Bias Assessment

**Figure 10:**
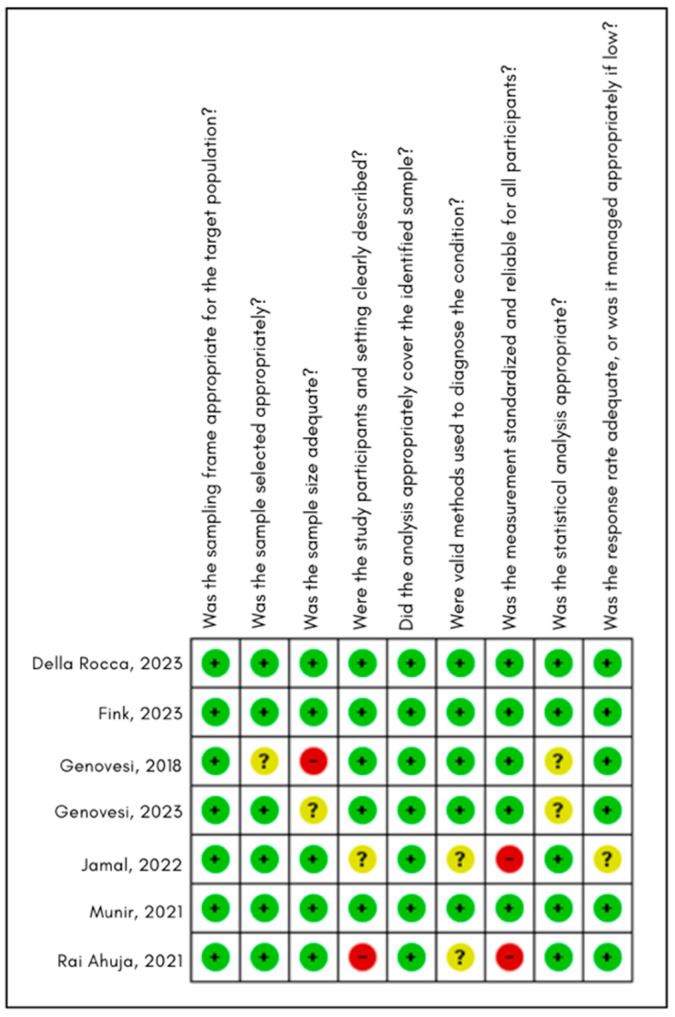
Risk of bias assessment for the included studies using the Munn et al. tool for prevalence data. Green indicates low risk of bias, yellow indicates unclear risk, and red indicates high risk across the nine evaluated domains [33,35,36,37,38,39,44].

In the individual assessment matrix (Figure 10), most studies showed a low risk of bias in key domains such as adequacy of the sampling frame, participant selection, context description, and the use of appropriate diagnostic methods.

Some limitations were identified: in two studies, the sample size was considered insufficient, and in others, a high risk was observed in the domain related to the validity of diagnostic methods due to incomplete information.

**Figure 11:**
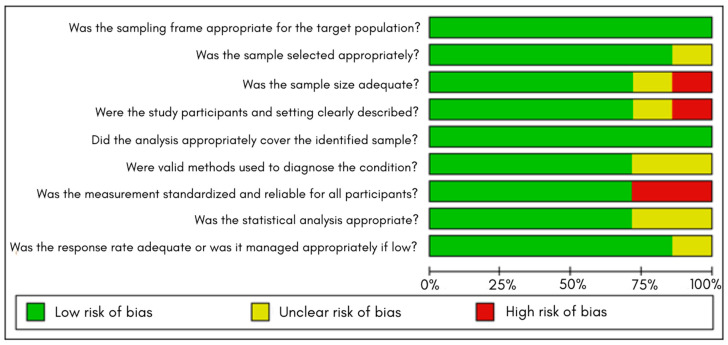
Summary of the risk of bias assessment across all included studies using the Munn et al. tool. Bars represent the proportion of studies rated as low, unclear, or high risk of bias for each methodological domain.

The summary figure (Figure 11) shows that, overall, the studies demonstrate good methodological quality, with few areas of risk that do not meaningfully compromise the robustness of the meta-analysis results.

## 5. Discussion

This systematic review and meta-analysis represents, to the best of current evidence, the first attempt to quantitatively summarize the frequency of thromboembolic events, bleeding, and mortality in patients with AF and ESRD undergoing LAAC, a population with extremely high clinical risk in whom conventional anticoagulation poses a persistent therapeutic dilemma due to the simultaneous propensity for stroke and major bleeding. The pooled frequencies were 3% for thromboembolic events, 6% for bleeding, and 2% for overall mortality, with considerable heterogeneity across studies. These findings suggest that LAAC may offer protection against embolic events with an acceptable bleeding profile, even in a population with advanced multiorgan dysfunction and clinical frailty. Mortality, although not negligible, appears more closely related to the comorbidity burden inherent to ESRD rather than to the procedure itself.

Compared with conventional antithrombotic therapies, these findings are notable. In the network meta-analysis by Kao et al. [48], which included 42 studies and 185,864 patients with AF and ESRD, the pooled thromboembolic event rates were 4.64% with VKAs (Supplementary Figure S1), 2.66% with DOACs (Supplementary Figure S2), and 7.77% among non-anticoagulated patients (Supplementary Figure S3), all substantially higher than the rate observed in our analysis, supporting the possibility that LAAC offers embolic protection comparable or superior to long-term anticoagulation without its associated bleeding risk. Similarly, the bleeding rate identified in this review (6%) was comparable to or lower than that reported with VKAs or DOACs (5.8% in both groups; Supplementary Figures S4 and S5) and clearly lower than that in non-anticoagulated patients (9.14%; Supplementary Figure S6). Moreover, the pooled mortality (2%) was markedly lower than that described in cohorts treated with anticoagulation or without antithrombotic therapy (16.01%, 23.76%, and 81.25%, respectively; Supplementary Figures S7–S9), suggesting an overall survival benefit associated with LAAC [49,50,51,52,53,54,55,56,57,58,59,60,61,62,63,64,65,66,67,68,69,70,71,72,73,74,75,76]. Recent procedural evidence also suggests that device overcompression during Watchman FLX implantation may reduce the incidence of residual peridevice leak without increasing procedural complications, highlighting the importance of technical optimization during LAAC implantation [77].

Previous reviews have assessed the safety and efficacy of LAAC in AF and CKD but have primarily focused on comparing patients with and without renal dysfunction, without direct analyses against oral anticoagulation [40,78,79,80,81,82]. Consistently, these studies show that ESRD represents a high-risk cohort, yet LAAC maintains comparable efficacy in preventing cardioembolic events. Higher mortality and bleeding rates in ESRD groups are attributed to comorbidity burden rather than the procedure itself. Rodríguez et al. and Gill et al. [83,84] reported similar incidences of thromboembolic events and device-related thrombosis between ESRD and non-ESRD groups, with higher mortality and bleeding in ESRD due to increased baseline risk factors.

The study by Genovesi et al. [38], was the only one to directly compare LAAC versus VKAs or no therapy, demonstrating a significant reduction in thromboembolic events (HR 0.19; 95% CI 0.04–0.96; *p* = 0.045) and (HR 0.16; 95% CI 0.04–0.66; *p* = 0.011) and bleeding compared with VKAs (HR 0.37; 95% CI 0.16–0.83; *p* = 0.017) [39]. Similarly, Dhar et al. [83] found lower mortality (HR 0.47; 95% CI, 0.3–0.72) and reduced recurrent bleeding (HR 0.74; 95% CI, 0.56–0.98) with LAAC compared with anticoagulants, with no increase in stroke. This alignment with the findings of the present meta-analysis reinforces the potential of LAAC as an effective and safe alternative for patients with ESRD.

Collectively, this review provides a novel quantitative estimate of clinically relevant events in a historically underrepresented population, expanding the understanding of the benefit/risk profile of LAAC and suggesting a potential shift in the management approach to AF in ESRD when systemic anticoagulation is risky or ineffective.

## 6. Limitations of the Evidence Included

Interpretation of the findings of this review must consider several limitations inherent to the available primary evidence. First, most of the included studies were observational, predominantly retrospective or non-randomized prospective cohorts, and without random allocation or blinding, introducing the risk of selection bias and confounding by indication. Patients selected for LAAC may have had more favorable clinical characteristics, such as lower frailty or greater hemodynamic stability, compared with those who remained on medical anticoagulation or without treatment, potentially influencing the outcomes observed.

Additionally, some studies relied on administrative databases or national registries (such as NIS or NRD), which lack detailed clinical information, including laboratory parameters, anticoagulation status before or at discharge, technical procedural characteristics, device type used, or the presence of device-related thrombosis. The absence of these data limits the ability to perform robust multivariable adjustments and to identify predictors of clinical events.

Long-term follow-up varied substantially across studies, from days to several years, which may have influenced cumulative rates of thromboembolic events, bleeding, and mortality. Likewise, not all studies systematically reported periprocedural events or those occurring outside the hospital setting, introducing the possibility of underreporting complications.

The sample size of the ESRD subgroup was small even in large-scale studies, resulting in statistical imprecision and wide confidence intervals in the estimates. This limitation reduces the power to detect significant differences and may partially explain the high heterogeneity (elevated I^2^ values) observed in the pooled analyses.

Moreover, the methodological quality of the studies was variable. Although most met acceptable standards according to the STROBE checklist, inconsistencies were identified in the reporting of variables and outcomes, as well as the absence of sensitivity analyses or adjustment for relevant confounders. The lack of randomized clinical trials remains the primary limitation of the current evidence. Previous efforts to fill this gap, such as the Watch-AFIB and STOP-HARM trials, were prematurely halted due to recruitment challenges, highlighting the logistical and ethical difficulties of evaluating LAAC in dialysis patients.

Finally, there was considerable clinical and methodological heterogeneity among the included studies. Differences in inclusion criteria, associated comorbidities, device types (Watchman™, Amulet™, others), and post-implant antithrombotic management protocols may have contributed to variability in outcomes. This heterogeneity, although expected in such a complex population, reduces the precision of pooled estimates and limits the generalizability of the findings.

Taken together, these limitations suggest that the overall certainty of the evidence regarding LAAC in patients with AF and ESRD should be considered low according to GRADE criteria. While the observed results are coherent and clinically plausible, confidence in the estimates is restricted by the observational nature of the data and the absence of high-quality randomized trials.

## 7. Limitations of the Review Process

Despite the methodological rigor applied, this review presents several limitations inherent to the review process itself. Although the search strategy was extensive, the possibility of publication bias remains, as studies with negative, neutral, or small-sample results may not have been published or indexed. There is also a potential language and availability bias, since only studies with accessible full-text articles were included, which may have excluded some of the relevant literature, particularly from Asian countries and Eastern Europe.

The quantitative synthesis was limited by substantial heterogeneity (I^2^ > 75% for some outcomes), which restricted the ability to perform subgroup analyses or meta-regressions. The observed heterogeneity, particularly for mortality (I^2^ = 97.8%) and bleeding (I^2^ = 75.2%), reflects marked differences in baseline characteristics among ESRD patients, including age, comorbidities, time on dialysis, cardiovascular status, renal replacement modality, and residual kidney function, all of which directly influence event risk. Variability in study designs and follow-up durations also contributed, with shorter studies capturing mainly periprocedural complications, whereas longer studies reflected cumulative events. Additional differences in technical expertise across participating centers further increased heterogeneity.

Clinical outcomes were defined and reported heterogeneously, using variable criteria (BARC, TIMI, ISTH) and with inconsistent reporting of major and/or minor events. Small sample sizes in several studies increased statistical instability and amplified I^2^ values. The predominance of observational designs, heterogeneous adjustment for confounders, and lack of randomization introduced further residual variability. Collectively, these factors explain the high heterogeneity observed and justify the use of a random-effects model to obtain more conservative estimates.

Minor modifications were implemented, such as extending the search timeframe to September 2024 and adding institutional repositories, without altering the predefined inclusion criteria or methodological framework, although these adjustments may have introduced minimal variability in the identification of sources. The process remained consistent with PRISMA 2020, ensuring systematic and reproducible search and selection procedures.

The methodology employed strengthened the reliability of the findings: an exhaustive search without unnecessary restrictions; independent selection and data extraction by reviewers with consensus resolution; use of validated quality appraisal tools; and application of appropriate statistical techniques, including random-effects models. These measures contributed to methodological rigor and coherence; however, the limitations of both the primary evidence and the review process should be considered when interpreting the magnitude and generalizability of the results.

## 8. Implications for Clinical Practice, Policy, and Research

The findings of this review have important clinical and scientific implications for the management of patients with AF and ESRD, in whom oral anticoagulation represents a major challenge due to the coexistence of high thromboembolic and bleeding risks. The results suggest that percutaneous LAAC may be a viable and safe alternative in selected patients, offering embolic protection without the increased bleeding risk associated with anticoagulation. The low combined frequency of stroke and bleeding supports the plausibility of its use in patients with contraindications to anticoagulation or a history of major hemorrhage, a common scenario in those receiving hemodialysis. LAAC emerges as a non-pharmacological option for stroke prevention when medical therapy is inadequate or hazardous.

These findings support a multidisciplinary approach involving cardiologists, electrophysiologists, and nephrologists to evaluate thrombotic and bleeding risks, with careful patient selection, particularly for those with high CHA_2_DS_2_-VASc and HAS-BLED scores and elevated Charlson comorbidity index [84]. Implementing institutional protocols that address preprocedural preparation, post-implant antithrombotic management, and structured echocardiographic follow-up may optimize outcomes and reduce complications. For healthcare systems, LAAC could reduce hospitalizations, disabling embolic events, and major hemorrhages, ultimately improving quality of life and resource utilization. At the policy level, these data justify considering LAAC within future clinical guidelines for AF and ESRD patients who are not candidates for anticoagulation. Although current evidence is moderate, the benefit–risk profile supports its evaluation by major scientific societies such as the ESC, ACC/AHA/HRS, and KDIGO

Future research should address the existing gaps, underscoring the need for higher-quality studies, preferably randomized clinical trials or well-designed prospective comparative studies evaluating LAAC versus conventional anticoagulation or conservative management in ESRD. These studies should focus on hard outcomes such as cardiovascular mortality, disabling stroke, and major bleeding, and should include subgroup analyses to identify patient profiles that benefit most. Additional research is needed to compare different devices, define post-implant antithrombotic strategies tailored to residual renal function, and assess long-term durability of occlusion. Evaluating quality of life, reductions in hospitalizations, and cost-effectiveness would provide key insights. Establishing multicenter registries specifically for AF and ESRD would generate more robust and representative evidence to inform future guidelines.

In conclusion, the findings support the feasibility, safety, and potential efficacy of LAAC as a promising intervention for managing thromboembolic risk in patients with AF and ESRD. Although widespread adoption should be approached cautiously and on an individualized basis, current evidence points to its potential to fill a clinically important therapeutic gap in a population with limited pharmacologic options. Confirming these results through high-quality prospective studies will be essential to define its definitive role in clinical practice.

## Figures and Tables

**Table 1:** General characteristics of the included studies (N = 2571). This table summarizes the country, study design, sample size (*n*), proportion of patients on dialysis, age (mean ± SD or median [IQR]), and sex distribution for each study included in the scoping review.

Study	Ahuja 2021 [34]	Areiza, 2024 [44]	Besabe, 2024 [45]	Della Rocca, 2023 [33]	Fastner, 2021 [46]	Fink, 2023 [35]	Genovesi, 2018 [39]	Genovesi, 2023 [38]	Jamal, 2022 [36]	Kefer, 2016 [47]	Manes, 2020 [41]	Munir, 2021 [37]	Ueno, 2023 [42]	Xipell, 2019 [43]
n	571	17	8	65	15	57	50	106	465	19	6	115	25	8
Following time	90	377.1	441 ± 231	390 (180–600)	365	500	30	1460	2	498 (186–660)	430 (90–660)	1 (1–1)	45	427.2 ± 283.3
Woman (%)	34.4	59	12.5	20.2	20	29.8	24	27.4	31.2	42.11	NR	32.3	20	25
Age	69.53 ± 20.87	66.9 ± 8.4	78.8 ± 6.7	76.8 (69.1–81.4)	75 (69–79)	73.9 ± 7.4	71.8	74 (46–87)	69.9	76.9 ± 6.6	73	71 (65–78)	73 (71–79)	67.5 ± 7.2
BMI	NR	NR	NR	28.1 (23.3–31.7)	25 (23–32)	28.5 ± 7	25.5	25 (22–28)	NR	27.6 ± 5	NR	NR	21.6 (20–24.4)	26.56
Baseline Comorbidities														
Hypertension	351	15	7	68	14	54	13	95	NR	13	6	724	16	8
Diabetes Mellitus	333	9	2	40	10	30	12	40	270	8	1	40	12	3
Heart Failure	297	5	3	40	6	27	21	36	230	7	NR	585	9	2
Hyperlipidemia	304	NR	7	NR	NR	NR	22	40	250	NR	NR	NR	18	3
Previous Stroke	111	6	1	20	3	10	7	11	90	9	0	NR	15	2
Previous TIA	NR	NR	NR	8	1	6	NR	NR	NR	NR	0	NR	1	NR
Previous bleeding	NR	27	6	NR	14	20	31	66	NR	13	NR	NR	7	8
Minor bleeding	NR	NR	NR	4	NR	NR	NR	NR	NR	NR	NR	NR	NR	NR
Major bleeding	NR	14	2	40	6	NR	31	66	NR	13	NR	NR	NR	NR
Acid peptic disease	NR	NR	NR	28	NR	NR	NR	NR	NR	NR	NR	NR	NR	NR
Hepatic disease	9	NR	NR	15	NR	NR	NR	NR	20	NR	1	65	0	2
Smoker	182	NR	4	NR	NR	NR	10	10	170	NR	NR	NR	NR	NR
CHA2DS2VASC (mean ± SD or median [IQR])	3.81 ±1.4	3 (2–3)	4.8 ± 1.5	5 (4–6)	5.1 ± 1.7	4.6	4	4 (3–5)	NR	4.7 ± 1.7	4.1	3 (2–4)	5 (4–6)	4.75 ± 1.16
HASBLED (mean ± SD or median [IQR])	NR	4 (4–4)	3.8 ± 1.3	4 (4–5)	4.6 ± 1.1	3.5	4.4	4 (4–5)	NR	4.3 ± 1.3	5.8	NR	4 (3–5)	4.62 ± 0.91
AF phenotype														
Permanent	NR	NR	2	NR	5	NR	27	50	NR	14	2	NR	NR	NR
Persistent	NR	NR	NR	NR	3	NR	7	18	NR	NR	3	NR	NR	NR
Paroxysmal	NR	NR	NR	NR	7	NR	16	38	NR	N5	NR	NR	NR	NR
Device														
Watchman	NR	NR	8	69	NR	26	24	NR	NR	NR	6	NR	25	3
Amulet	NR	NR	0	0	NR	NR	0	NR	NR	NR	0	NR	NR	NR
Anticoagulant medication	NR	15	6	NR	9	7	18	31	180	3	6	NR	NR	NR
Antiplatelet medication	NR	NR	2	NR	6	49	40	NR	NR	NR	NR	NR	NR	8
Periprocedural Complications														
Device embolism	2	0	0	0	0	0	0	NR	NR	0	0	NR	0	NR
Bleeding	24	0	1	1	1	4	0	NR	NR	0	0	44	1	0
Infection	NR	NR	NR	NR	0	NR	NR	NR	NR	NR	NR	NR	NR	NR

Abbreviations: SD = standard deviation; IQR = interquartile range; BMI: body mass index; AF: atrial fibrillation; TIA: transient ischemic attack. Source: Authors’ own elaboration based on data synthesis from the included studies.

## Data Availability

The data that support the findings of this study are available on request from the corresponding author.

## Supplementary Materials

The following supporting information can be downloaded at: https://www.mdpi.com/article/10.3390/jcm15072641/s1, Figure S1: Frequency of thromboembolic events in patients with atrial fibrillation and end-stage renal disease anticoagulated with warfarin. Figure S2: Frequency of thromboembolic events in patients with atrial fibrillation and end-stage renal disease anticoagulated with DOAC. Figure S3: Frequency of thromboembolic events in patients with atrial fibrillation and end-stage renal disease without anticoagulation. Figure S4: Frequency of bleeding in patients with atrial fibrillation and end-stage renal disease anticoagulated with warfarin. Figure S5: Frequency of bleeding in patients with atrial fibrillation and end-stage renal disease anticoagulated with DOAC. Figure S6: Frequency of bleeding in patients with atrial fibrillation and end-stage renal disease anticoagulated without anticoagulation. Figure S7: Frequency of all-cause mortality in patients with atrial fibrillation and end-stage renal disease anticoagulated with warfarin. Figure S8: Frequency of all-cause mortality in patients with atrial fibrillation and end-stage renal disease anticoagulated with DOAC. Figure S9: Frequency of all-cause mortality in patients with atrial fibrillation and end-stage renal disease without anticoagulation. Table S1: PRISMA 2020 Checklist. Table S2: Detailed Search Strategy. Table S3: Detailed Eligibility Criteria.
