# Supplementary material for: Systematic Review and Meta-Analysis of the Frequency of Thromboembolic Events, Bleeding, and Mortality in Patients with Atrial Fibrillation and End-Stage Renal Disease Undergoing Percutaneous Left Atrial Appendage Closure
Source: J Clin Med. 2026 Mar 31;15(7):2641. doi: 10.3390/jcm15072641 (PMC13073027; doi:10.3390/jcm15072641)
Supplement: Supplementary file 1 [file jcm-15-02641-s001.zip › jcm-4194281-supplementary.pdf]

Supplementary Materials

Table S1. PRISMA 2020 Checklist

Section and Topic	Item #	Checklist item	Location where item is reported
**TITLE**			
Title	1	Identify the report as a systematic review.	1
**ABSTRACT**			
Abstract	2	See the PRISMA 2020 for Abstracts checklist.	1
**INTRODUCTION**			
Rationale	3	Describe the rationale for the review in the context of existing knowledge.	2-5
Objectives	4	Provide an explicit statement of the objective(s) or question(s) the review addresses.	5
**METHODS**			
Eligibility criteria	5	Specify the inclusion and exclusion criteria for the review and how studies were grouped for the syntheses.	5-6
Information sources	6	Specify all databases, registers, websites, organisations, reference lists and other sources searched or consulted to identify studies. Specify the date when each source was last searched or consulted.	5
Search strategy	7	Present the full search strategies for all databases, registers and websites, including any filters and limits used.	5-6
Selection process	8	Specify the methods used to decide whether a study met the inclusion criteria of the review, including how many reviewers screened each record and each report retrieved, whether they worked independently, and if applicable, details of automation tools used in the process.	5-6
Data collection process	9	Specify the methods used to collect data from reports, including how many reviewers collected data from each report, whether they worked independently, any processes for obtaining or confirming data from study investigators, and if applicable, details of automation tools used in the process.	2
Data items	10a	List and define all outcomes for which data were sought. Specify whether all results that were compatible with each outcome domain in each study were sought (e.g. for all measures, time points, analyses), and if not, the methods used to decide which results to collect.	5
	10b	List and define all other variables for which data were sought (e.g. participant and intervention characteristics, funding sources). Describe any assumptions made about any missing or unclear information.	5
Study risk of bias assessment	11	Specify the methods used to assess risk of bias in the included studies, including details of the tool(s) used, how many reviewers assessed each study and whether they worked independently, and if applicable, details of automation tools used in the process.	7
Effect measures	12	Specify for each outcome the effect measure(s) (e.g. risk ratio, mean difference) used in the synthesis or presentation of results.	7
Synthesis methods	13a	Describe the processes used to decide which studies were eligible for each synthesis (e.g. tabulating the study intervention characteristics and comparing against the planned groups for each synthesis (item #5)).	7-8
	13b	Describe any methods required to prepare the data for presentation or synthesis, such as handling of missing summary statistics, or data conversions.	7-8
	13c	Describe any methods used to tabulate or visually display results of individual studies and syntheses.	7-8
	13d	Describe any methods used to synthesize results and provide a rationale for the choice(s). If meta-analysis was performed, describe the model(s), method(s) to identify the presence and extent of statistical heterogeneity, and software package(s) used.	8
	13e	Describe any methods used to explore possible causes of heterogeneity among study results (e.g. subgroup analysis, meta-regression).	8
	13f	Describe any sensitivity analyses conducted to assess robustness of the synthesized results.	8
Reporting bias assessment	14	Describe any methods used to assess risk of bias due to missing results in a synthesis (arising from reporting biases).	8
Certainty assessment	15	Describe any methods used to assess certainty (or confidence) in the body of evidence for an outcome.	8
**RESULTS**			
Study selection	16a	Describe the results of the search and selection process, from the number of records identified in the search to the number of studies included in the review, ideally using a flow diagram.	28
	16b	Cite studies that might appear to meet the inclusion criteria, but which were excluded, and explain why they were excluded.	28
Study characteristics	17	Cite each included study and present its characteristics.	27
Risk of bias in studies	18	Present assessments of risk of bias for each included study.	33
Results of individual studies	19	For all outcomes, present, for each study: (a) summary statistics for each group (where appropriate) and (b) an effect estimate and its precision (e.g. confidence/credible interval), ideally using structured tables or plots.	11,30

Section and Topic	Item #	Checklist item	Location where item is reported
Results of syntheses	20a	For each synthesis, briefly summarise the characteristics and risk of bias among contributing studies.	28
	20b	Present results of all statistical syntheses conducted. If meta-analysis was done, present for each the summary estimate and its precision (e.g. confidence/credible interval) and measures of statistical heterogeneity. If comparing groups, describe the direction of the effect.	27
	20c	Present results of all investigations of possible causes of heterogeneity among study results.	27
	20d	Present results of all sensitivity analyses conducted to assess the robustness of the synthesized results.	30
Reporting biases	21	Present assessments of risk of bias due to missing results (arising from reporting biases) for each synthesis assessed.	33
Certainty of evidence	22	Present assessments of certainty (or confidence) in the body of evidence for each outcome assessed.	30
**DISCUSSION**			
Discussion	23a	Provide a general interpretation of the results in the context of other evidence.	14,15
	23b	Discuss any limitations of the evidence included in the review.	15
	23c	Discuss any limitations of the review processes used.	17
	23d	Discuss implications of the results for practice, policy, and future research.	18
**OTHER INFORMATION**			
Registration and protocol	24a	Provide registration information for the review, including register name and registration number, or state that the review was not registered.	5
	24b	Indicate where the review protocol can be accessed, or state that a protocol was not prepared.	5
	24c	Describe and explain any amendments to information provided at registration or in the protocol.	5
Support	25	Describe sources of financial or non-financial support for the review, and the role of the funders or sponsors in the review.	Yes
Competing interests	26	Declare any competing interests of review authors.	Yes
Availability of data, code and other materials	27	Report which of the following are publicly available and where they can be found: template data collection forms; data extracted from included studies; data used for all analyses; analytic code; any other materials used in the review.	NA

Table S2. Detailed Search Strategy.

PubMed

	Search lines	HITS
**#1**	("Atrial Fibrillation"[Title] OR "atrial appendage"[MeSH Terms] OR "atrial appendage occlusion"[Title/Abstract] OR "atrial appendage closure"[Title/Abstract] OR "atrium appendage"[Title/Abstract] OR "auricular appendage"[Title/Abstract] OR "watchman"[Title/Abstract] OR "amulet"[Title/Abstract])	62,296
**#2**	("renal insufficiency, chronic"[MeSH Terms] OR "hemodialysis"[Title/Abstract] OR "chronic kidney"[Title/Abstract] OR "ESRD"[Title/Abstract] OR "end-stage renal disease"[Title/Abstract])	235,700
**#3**	**#1 AND #2**	1,302
**#4**	"prognost*"[Title/Abstract] OR "effectiv*"[Title/Abstract] OR "safe*"[Title/Abstract] OR "outcom*"[Title/Abstract] OR "stroke"[Title/Abstract] OR "bleed*"[Title/Abstract] OR "death"[Title/Abstract]	6,940,682
**#5**	**#3 AND #4**	955

BVSalud

	Search lines	HITS
**#1**	(chronic renal insufficiency OR hemodialysis OR chronic kidney OR end-stage renal disease) AND (atrial appendage OR auricular appendage) AND (db:("IBECS" OR "WPRIM" OR "LILACS" OR "AIM" OR "SES-SP"))	9

Embase.

	Search lines	HITS
#1	'chronic kidney failure'/exp OR 'chronic kidney failure' OR 'hemodialysis'/exp OR 'hemodialysis' OR 'end stage renal disease'/exp OR 'end stage renal disease' OR 'chronic kidney failure'/exp OR 'chronic kidney failure'	434,413
#2	'heart atrium appendage'/exp OR 'heart atrium appendage' OR 'atrial appendage occlusion' OR 'atrial appendage closure' OR 'watchman' OR 'amulet'	17,290
#3	#1 AND #2 AND [embase]/lim NOT ([embase]/lim AND [medline]/lim)	261

EBM Reviews & Cochrane Library (Via OVID)

	Search lines	HITS
**#1**	"chronic renal insufficiency".ab. or "chronic renal insufficiency ".hw. or "chronic renal".ti. OR "chronic kidney failure".ab. or "chronic kidney failure".hw. or "chronic kidney".ti. OR "hemodialysis".ab. or "hemodialysis".hw. or "hemodialysis".ti. OR "end stage renal disease".ab. or "end stage renal disease".hw. or "end stage renal disease".ti.	25,200
**#2**	"atrial appendage".ab. or "atrial appendage".hw. or "atrial appendage".ti. OR "atrium appendage".ab. or "atrium appendage".hw. or "atrium appendage".ti. OR "auricular appendage".ab. or "auricular appendage".hw. or "auricular appendage".ti. OR "watchman".ab. or "watchman".ti. OR "amulet".ab. or "amulet".ti.	796
**#3**	**#1 AND #2**	**12**

Texas Data Repository

	Queries	HITS
**#1**	dvDescription:"chronic renal insufficiency" OR (dsDescriptionValue:"chronic renal insufficiency" AND keywordValue:"chronic renal insufficiency")	0
**#2**	dvDescription:"atrial appendage" OR keywordValue:"atrial appendage"	0

https://dataverse.tdl.org/dataverse/root/?q=dvDescription%3A%22chronic+renal+insufficiency%22+OR+%28dsDescriptionValue%3A%22chronic+renal+insufficiency%22+AND+keywordValue%3A%22chronic+renal+insufficiency%22%29.

https://dataverse.tdl.org/dataverse/root/?q=dvDescription%3A%22atrial+appendage%22+OR+keywordValue%3A%22atrial+appendage%22.

Electrophysiology Physiome Model Repository

	Queries	HITS
**#1**	“chronic renal insufficiency"	0
**#2**	"chronic kidney"	2
**#3**	"atrial appendage”	2

1.https://models.physiomeproject.org/@@search?SearchableText=chronic+renal+insufficiency.

2. https://models.physiomeproject.org/@@search?SearchableText=chronic+kidney.

3. https://models.physiomeproject.org/@@search?SearchableText=atrial+appendage.

Radbpund University Repository.

	Queries	HITS
**#1**	Subject: "chronic renal insufficiency" **AND** Subject: "atrial appendage" ×	0
**#2**	Title: "chronic renal" **AND** Title: "atrial appendage" ×	0
**#3**	Subject: "chronic kidney"	6
**#4**	Title: "atrial appendage"	5

https://repository.ubn.ru.nl/discover?filtertype_1=subject&filter_relational_operator_1=contains&filter_1=%22chronic+renal+insufficiency%22&filtertype_2=subject&filter_relational_operator_2=contains&filter_2=%22atrial+appendage%22&submit_apply_filter=.

https://repository.ubn.ru.nl/discover?filtertype_1=title&filter_relational_operator_1=contains&filter_1=%22chronic+renal%22&filtertype_2=title&filter_relational_operator_2=contains&filter_2=%22atrial+appendage%22&submit_apply_filter=.

https://repository.ubn.ru.nl/discover?filtertype_1=subject&filter_relational_operator_1=contains&filter_1=%22chronic+kidney%22&submit_apply_filter=.

https://repository.ubn.ru.nl/discover?filtertype_1=title&filter_relational_operator_1=contains&filter_1=%22atrial+appendage%22&submit_apply_filter=.

Universidad del Rosario Institutional Repository.

	Queries	HITS
**#1**	"falla renal cronica" AND "cierre percutaneo"	0
**#2**	"cierre percutaneo"	12
**#3**	"falla renal cronica"	39
**#4**	**"Enfermedad renal crónica" AND "cierre percutaneo"**	**2**

https://repository.urosario.edu.co/search?query=%22falla%20renal%20cronica%22.

https://repository.urosario.edu.co/search?query=%22cierre%20percutaneo%22.

https://repository.urosario.edu.co/search?query=%20%22Enfermedad%20renal%20cr%C3%B3nica%22%20AND%20%22cierre%20percut%C3%A1neo%22.

Table S3. Detailed Eligibility Criteria.

Inclusion Criteria	Exclusion Criteria
Original articles	Studies in which the reported information did not allow the calculation of absolute and relative frequencies.
Articles in any language	Studies with inconsistencies in numerical data.
Articles available in the databases consulted up to September 2024	Studies that did not provide ESRD-specific stratified information.
Articles reporting absolute and relative frequencies	
Articles including patients with a prior diagnosis of AF and a diagnosis of ESRD, defined as an eGFR <15 mL/min/1.73 m² or requiring dialysis	

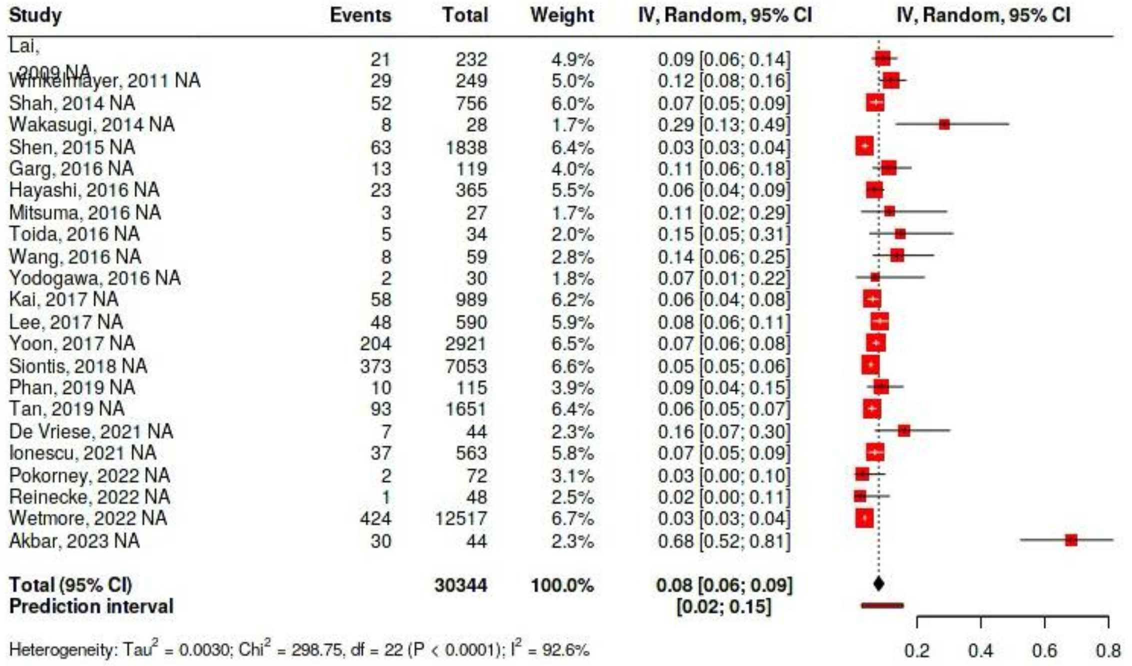

Figure S1. Frequency of thromboembolic events in patients with atrial fibrillation and end-stage renal disease anticoagulated with warfarin.

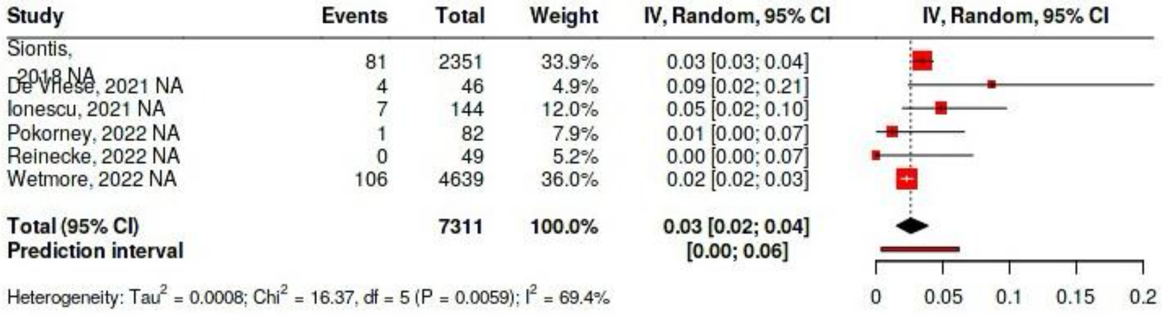

Figure S2. Frequency of thromboembolic events in patients with atrial fibrillation and end-stage renal disease anticoagulated with DOAC.

Study	Events	Total	Weight	IV, Random, 95% CI
Lai, 2009 NA	43	167	6.1%	0.26 [0.19; 0.33]
Winkelmayer, 2011 NA	135	2064	7.2%	0.07 [0.06; 0.08]
Shah, 2014 NA	55	870	7.0%	0.06 [0.05; 0.08]
Wakasugi, 2014 NA	5	32	3.6%	0.16 [0.05; 0.33]
Shen, 2015 NA	503	10446	7.3%	0.05 [0.04; 0.05]
Garg, 2016 NA	21	183	6.2%	0.11 [0.07; 0.17]
Hayashi, 2016 NA	43	692	7.0%	0.06 [0.05; 0.08]
Mitsuma, 2016 NA	5	55	4.5%	0.09 [0.03; 0.20]
Toida, 2016 NA	13	124	5.7%	0.10 [0.06; 0.17]
Wang, 2016 NA	11	82	5.2%	0.13 [0.07; 0.23]
Yodogawa, 2016 NA	5	54	4.5%	0.09 [0.03; 0.20]
Kai, 2017 NA	88	3297	7.2%	0.03 [0.02; 0.03]
Lee, 2017 NA	151	5150	7.3%	0.03 [0.02; 0.03]
Yoon, 2017 NA	201	7053	7.3%	0.03 [0.02; 0.03]
Phan, 2019 NA	11	361	6.7%	0.03 [0.02; 0.05]
Tan, 2019 NA	644	4114	7.3%	0.16 [0.15; 0.17]
**Total (95% CI)**		**34744**	**100.0%**	**0.08 [0.05; 0.10]**
**Prediction interval**				**[0.01; 0.21]**

Heterogeneity: $Tau^2 = 0.0074$; $Chi^2 = 851.86$, df = 15 ($P < 0.0001$); $I^2 = 98.2\%$

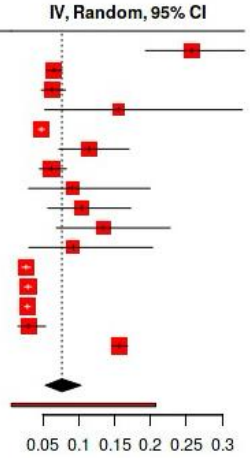

Figure S3. Frequency of thromboembolic events in patients with atrial fibrillation and end-stage renal disease without anticoagulation.

Study	Events	Total	Weight	IV, Random, 95% CI
Winkelmayer, 2011 0	11	249	4.4%	0.04 [0.02; 0.08]
Shah, 2014 NA	149	756	4.6%	0.20 [0.17; 0.23]
Wakasugi, 2014 NA	3	28	2.8%	0.11 [0.02; 0.28]
Shen, 2015 NA	29	1838	4.7%	0.02 [0.01; 0.02]
Garg, 2016 NA	26	119	4.1%	0.22 [0.15; 0.30]
Hayashi, 2016 NA	60	365	4.5%	0.16 [0.13; 0.21]
Mitsuma, 2016 NA	7	27	2.8%	0.26 [0.11; 0.46]
Toida, 2016 NA	3	34	3.1%	0.09 [0.02; 0.24]
Wang, 2016 NA	22	59	3.6%	0.37 [0.25; 0.51]
Yodogawa, 2016 NA	3	30	2.9%	0.10 [0.02; 0.27]
Kai, 2017 NA	20	989	4.7%	0.02 [0.01; 0.03]
Lee, 2017 NA	8	590	4.6%	0.01 [0.01; 0.03]
Sarratt, 2017 NA	7	120	4.1%	0.06 [0.02; 0.12]
Yoon, 2017 NA	84	2921	4.7%	0.03 [0.02; 0.04]
Reed, 2018 NA	11	50	3.4%	0.22 [0.12; 0.36]
Siontis, 2018 NA	715	7053	4.7%	0.10 [0.09; 0.11]
Phan, 2019 NA	2	115	4.1%	0.02 [0.00; 0.06]
Tan, 2019 NA	407	1651	4.7%	0.25 [0.23; 0.27]
De Vriese, 2021 NA	24	44	3.3%	0.55 [0.39; 0.70]
Ionescu, 2021 NA	9	563	4.6%	0.02 [0.01; 0.03]
Pokorney, 2022 NA	7	72	3.8%	0.10 [0.04; 0.19]
Reinecke, 2022 NA	6	48	3.4%	0.12 [0.05; 0.25]
Wetmore, 2022 NA	1226	12517	4.7%	0.10 [0.09; 0.10]
Akbar, 2023 NA	3	44	3.3%	0.07 [0.01; 0.19]
Lai, 2009 NA	32	232	4.4%	0.14 [0.10; 0.19]
**Total (95% CI)**		**30514**	**100.0%**	**0.10 [0.08; 0.14]**
**Prediction interval**				**[0.00; 0.30]**

Heterogeneity: $Tau^2 = 0.0131$; $Chi^2 = 1241.92$, df = 24 ($P < 0.0001$); $I^2 = 98.1\%$

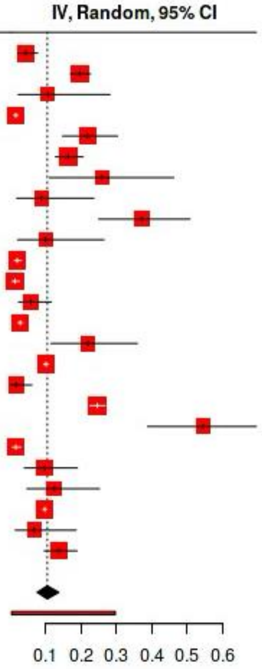

Figure S4. Frequency of bleeding in patients with atrial fibrillation and end-stage renal disease anticoagulated with warfarin.

Study	Events	Total	Weight	IV, Random, 95% CI
Reed, 2018 NA	4	74	12.1%	0.05 [0.01; 0.13]
Siontis, 2018 NA	129	2351	20.2%	0.05 [0.05; 0.06]
De Vriese, 2021 NA	21	46	9.6%	0.46 [0.31; 0.61]
Ionescu, 2021 NA	0	144	15.1%	0.00 [0.00; 0.03]
Pokorney, 2022 NA	9	82	12.6%	0.11 [0.05; 0.20]
Reinecke, 2022 NA	5	49	10.0%	0.10 [0.03; 0.22]
Wetmore, 2022 NA	244	4639	20.4%	0.05 [0.05; 0.06]
**Total (95% CI)**		**7385**	**100.0%**	**0.07 [0.04; 0.11]**
**Prediction interval**				**[0.00; 0.20]**

Heterogeneity: $Tau^2 = 0.0047$; $Chi^2 = 75.96$, df = 6 ($P < 0.0001$); $I^2 = 92.1\%$

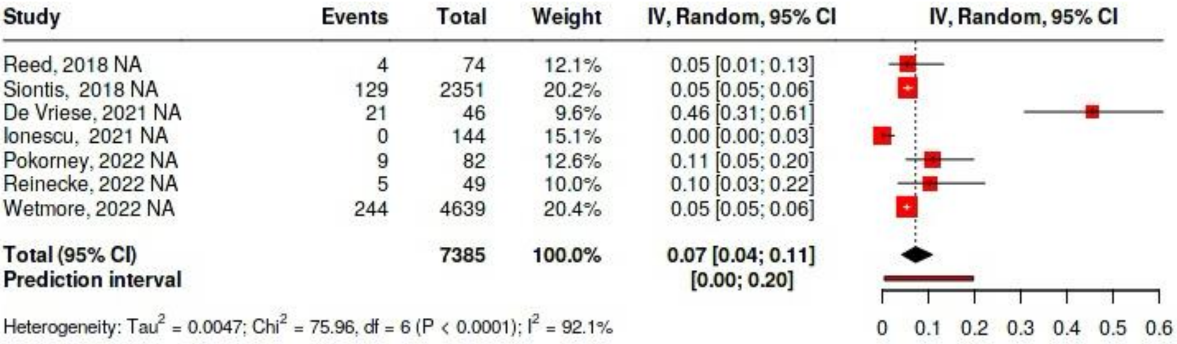

Figure S5. Frequency of bleeding in patients with atrial fibrillation and end-stage renal disease anticoagulated with DOAC.

Study	Events	Total	Weight	IV, Random, 95% CI
Winkelmayer, 2011 NA	21	2064	6.1%	0.01 [0.01; 0.02]
Shah, 2014 NA	126	870	6.1%	0.14 [0.12; 0.17]
Wakasugi, 2014 NA	4	32	5.2%	0.12 [0.04; 0.29]
Shen, 2015 NA	192	10446	6.1%	0.02 [0.02; 0.02]
Garg, 2016 NA	26	183	5.9%	0.14 [0.09; 0.20]
Hayashi, 2016 NA	54	692	6.1%	0.08 [0.06; 0.10]
Mitsuma, 2016 NA	9	55	5.5%	0.16 [0.08; 0.29]
Toida, 2016 NA	6	124	5.9%	0.05 [0.02; 0.10]
Wang, 2016 NA	24	82	5.7%	0.29 [0.20; 0.40]
Yodogawa, 2016 NA	1	54	5.5%	0.02 [0.00; 0.10]
Kai, 2017 NA	12	3297	6.1%	0.00 [0.00; 0.01]
Lee, 2017 NA	37	5150	6.1%	0.01 [0.01; 0.01]
Yoon, 2017 NA	50	7053	6.1%	0.01 [0.01; 0.01]
Phan, 2019 NA	3	361	6.0%	0.01 [0.00; 0.02]
Tan, 2019 NA	1559	4114	6.1%	0.38 [0.36; 0.39]
Akbar, 2023 NA	3	44	5.4%	0.07 [0.01; 0.19]
Lai, 2009 NA	15	167	5.9%	0.09 [0.05; 0.14]
**Total (95% CI)**		**34788**	**100.0%**	**0.07 [0.03; 0.13]**
**Prediction interval**				**[0.00; 0.44]**

Heterogeneity: $Tau^2 = 0.0429$; $Chi^2 = 4905.25$, df = 16 (P = 0); $I^2 = 99.7\%$

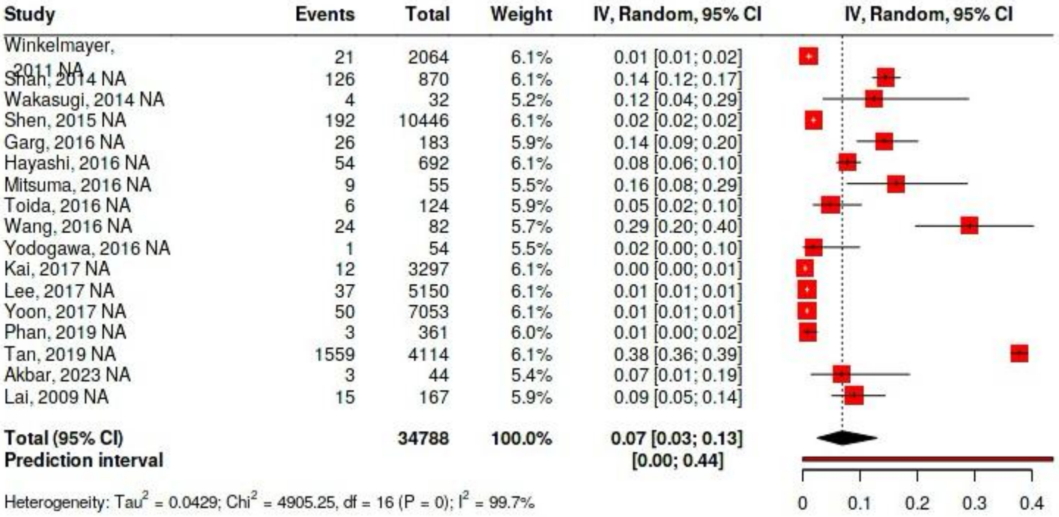

Figure S6. Frequency of bleeding in patients with atrial fibrillation and end-stage renal disease anticoagulated without anticoagulation.

Study	Events	Total	Weight	IV, Random, 95% CI
Chan, 2009 NA	333	749	5.0%	0.44 [0.41; 0.48]
Winkelmayer, 2011 NA	181	249	4.9%	0.73 [0.67; 0.78]
Wakasugi, 2014 NA	9	28	4.3%	0.32 [0.16; 0.52]
Shen, 2015 NA	832	1838	5.0%	0.45 [0.43; 0.48]
Brancaccio, 2016 NA	222	661	5.0%	0.34 [0.30; 0.37]
Garg, 2016 NA	97	119	4.8%	0.82 [0.73; 0.88]
Hayashi, 2016 NA	86	365	4.9%	0.24 [0.19; 0.28]
Mitsuma, 2016 NA	8	27	4.3%	0.30 [0.14; 0.50]
Wang, 2016 NA	44	59	4.6%	0.75 [0.62; 0.85]
Yodogawa, 2016 NA	6	30	4.3%	0.20 [0.08; 0.39]
Kai, 2017 NA	433	989	5.0%	0.44 [0.41; 0.47]
Lee, 2017 NA	350	590	5.0%	0.59 [0.55; 0.63]
Siontis, 2018 NA	753	7053	5.0%	0.11 [0.10; 0.11]
Voskamp, 2018 NA	141	244	4.9%	0.58 [0.51; 0.64]
Phan, 2019 NA	32	115	4.8%	0.28 [0.20; 0.37]
Tan, 2019 NA	476	1651	5.0%	0.29 [0.27; 0.31]
De Vriese, 2021 NA	32	44	4.5%	0.73 [0.57; 0.85]
Pokorney, 2022 NA	13	72	4.7%	0.18 [0.10; 0.29]
Reinecke, 2022 NA	12	48	4.6%	0.25 [0.14; 0.40]
Wetmore, 2022 NA	6096	12517	5.0%	0.49 [0.48; 0.50]
Akbar, 2023 NA	36	44	4.5%	0.82 [0.67; 0.92]
**Total (95% CI)**		**27492**	**100.0%**	**0.44 [0.34; 0.54]**
**Prediction interval**				**[0.05; 0.89]**

Heterogeneity: $Tau^2 = 0.0542$; $Chi^2 = 4285.85$, df = 20 (P = 0); $I^2 = 99.5\%$

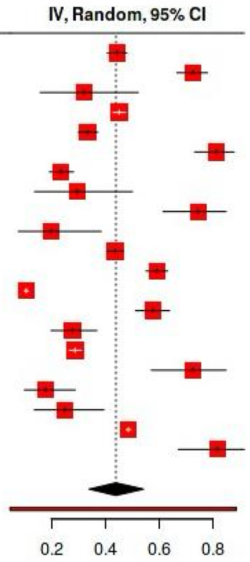

Figure S7. Frequency of all-cause mortality in patients with atrial fibrillation and end-stage renal disease anticoagulated with warfarin

Study	Events	Total	Weight	IV, Random, 95% CI
Siontis, 2018 NA	159	2351	20.9%	0.07 [0.06; 0.08]
De Vriese, 2021 NA	30	46	19.1%	0.65 [0.50; 0.79]
Pokorney, 2022 NA	21	82	19.9%	0.26 [0.17; 0.36]
Reinecke, 2022 NA	9	49	19.2%	0.18 [0.09; 0.32]
Wetmore, 2022 NA	1457	4639	21.0%	0.31 [0.30; 0.33]
**Total (95% CI)**		**7167**	**100.0%**	**0.27 [0.11; 0.47]**
**Prediction interval**				**[0.00; 0.91]**

Heterogeneity: $Tau^2 = 0.0531$; $Chi^2 = 724.89$, df = 4 ($P < 0.0001$); $I^2 = 99.4\%$

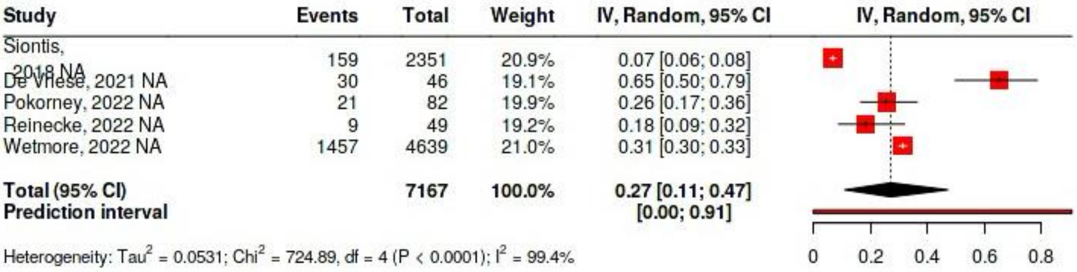

Figure S8. Frequency of all-cause mortality in patients with atrial fibrillation and end-stage renal disease anticoagulated with DOAC.

Study	Events	Total	Weight	IV, Random, 95% CI
Chan, 2009 NA	425	925	6.4%	0.46 [0.43; 0.49]
Winkelmayer, 2011 NA	750	2064	6.4%	0.36 [0.34; 0.38]
Wakasugi, 2014 NA	9	32	5.8%	0.28 [0.14; 0.47]
Shen, 2015 NA	4595	10446	6.4%	0.44 [0.43; 0.45]
Brancaccio, 2016 NA	250	2438	6.4%	0.10 [0.09; 0.12]
Garg, 2016 NA	145	183	6.3%	0.79 [0.73; 0.85]
Hayashi, 2016 NA	120	692	6.4%	0.17 [0.15; 0.20]
Mitsuma, 2016 NA	27	55	6.0%	0.49 [0.35; 0.63]
Wang, 2016 NA	64	82	6.1%	0.78 [0.68; 0.86]
Yodogawa, 2016 NA	15	54	6.0%	0.28 [0.16; 0.42]
Kai, 2017 NA	496	3297	6.4%	0.15 [0.14; 0.16]
Lee, 2017 NA	1051	5150	6.4%	0.20 [0.19; 0.22]
Voskamp, 2018 NA	540	1474	6.4%	0.37 [0.34; 0.39]
Phan, 2019 NA	98	361	6.3%	0.27 [0.23; 0.32]
Tan, 2019 NA	3349	4114	6.4%	0.81 [0.80; 0.83]
Akbar, 2023 NA	40	44	5.9%	0.91 [0.78; 0.97]
**Total (95% CI)**		**31411**	**100.0%**	**0.42 [0.30; 0.55]**
**Prediction interval**				**[0.02; 0.92]**

Heterogeneity: $Tau^2 = 0.0671$; $Chi^2 = 6933.03$, df = 15 (P = 0); $I^2 = 99.8\%$

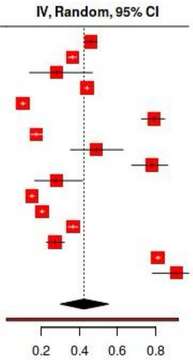

Figure S9. Frequency of all-cause mortality in patients with atrial fibrillation and end-stage renal disease without anticoagulation.
